# A computational continuum model of poroelastic beds

**DOI:** 10.1098/rspa.2016.0932

**Published:** 2017-03-22

**Authors:** U. Lācis, G. A. Zampogna, S. Bagheri

**Affiliations:** 1 Linné Flow Centre, Department of Mechanics KTH, 100 44 Stockholm, Sweden; 2 Institut de Mécanique des Fluides de Toulouse, Université de Toulouse, 31400 Toulouse, France

**Keywords:** poroelasticity, connected-structures, numerical simulation, anisotropy

## Abstract

Despite the ubiquity of fluid flows interacting with porous and elastic materials, we lack a validated non-empirical macroscale method for characterizing the flow over and through a poroelastic medium. We propose a computational tool to describe such configurations by deriving and validating a continuum model for the poroelastic bed and its interface with the above free fluid. We show that, using stress continuity condition and slip velocity condition at the interface, the effective model captures the effects of small changes in the microstructure anisotropy correctly and predicts the overall behaviour in a physically consistent and controllable manner. Moreover, we show that the performance of the effective model is accurate by validating with fully microscopic resolved simulations. The proposed computational tool can be used in investigations in a wide range of fields, including mechanical engineering, bio-engineering and geophysics.

## 1. Introduction

Recent advances in surface micro- and nano-fabrication techniques [1–3] are providing new technological opportunities of enormous potential. However, we lack high-fidelity models capturing how the underlying small-scale physico-chemical processes interact with the large-scale flow and heat- and mass-transport phenomena. The reason is the vast range of scales in both time and space that need be resolved in order to capture the full physical picture, which renders full-scale numerical investigations extremely costly [4]. The development of multi-scale models with reduced complexity is a necessary enabler in an increasing number of applications where the control of events at small length scales determines the properties of the flow over much larger space and time scales. Examples include, but are not limited to, the design of novel surfaces to control heat transfer, skin friction or pressure drag, acoustic noise and fluid mixing.

There is thus a clear need to develop techniques that combine computational fluid mechanics with mesoscopic or microscopic models of materials. In this work, we take a step in this direction by modelling the interaction of mesoscopic surface textures with macroscopic flows. By ‘mesoscopic’, we mean structures that can be described by continuum methods, but are still significantly smaller than the large-scale flow phenomena. When these constructions, which can take the form of high-aspect ratio structures (such as fibres) or granular-type structures (such as particles), are saturated with a fluid and can absorb the fluid stress and possibly deform, we have a poroelastic material.

The description of poroelastic medium as a continuum has an extensive and broad history, beginning with the empirical models of porous media by Darcy [5] and followed by the formulation of total stress tensor of deformable porous media by Biot [6,7]. Today, there exists a significant amount of work [8–28] on poroelastic media adopting top-down approaches based on empirical macroscale stress/deformation tests, bottom-up approaches using homogenization and volume averaging techniques, physical approaches using analytical and mechanical models or other methods.

In this work, we employ the method of homogenization via multi-scale expansion to model flows through and over poroelastic surfaces. Within the general multi-scale analysis platform (MAP) classification proposed by Scheibe *et al.* [29], the current method falls into the category of methods for which the different scales can be completely decoupled from each other (e.g. ‘motif B’ in [29]). In other words, our work is ‘formal upscaling’, which means that after the macroscale governing equations have been derived, the parameters of the model are governed by fully decoupled microscale problems (from now on, we use the term ‘microscale’ instead of ‘mesoscale’ to conform with the terminology within the two-scale expansion approach). This method limits our investigations to set-ups for which the underlying microscale closure problems are linear. For example, the fluid phase in the poroelastic material must be slow enough such that inertial effects can be neglected or modelled through some kind of linearization. Essentially all previous upscaling works on deformable porous media has been in the same motif B category. Examples include the method of volume averaging by Whitaker [30] and the method of homogenization, as employed, for example, by Mei & Vernescu [13].

There exists now a number of studies ([31] and references therein) on development and applications of the motif B multi-scale methods for poroelastic media. It is, however, the authors’ opinion that we still lack a computational framework that lays out the sequential steps needed to be taken in order to obtain the physical parameters describing the poroelastic medium not only in the interior of the material, but also at the interface with freely moving fluid. Ideally, such a framework, when provided a particular microscopic structure in terms of its geometry and properties (skeleton elasticity, connectivity, etc.), will provide the anisotropic macroscopic material properties (permeability, elasticity, etc.) of the effective continuum fields without any fitting parameters obtained from experiments.

To the best of the authors’ knowledge, the theoretical frameworks of Whitaker [14] and Mei & Vernescu [13] have not been validated from the microscopic point of view for the flow over a surface that is coated with porous and elastic material. Validations of these methods most often consider only macroscopic measures. More specifically, while microscale problems in unit cells needed in the upscaling procedures have been presented and solved previously [13,14,32], there is no comparison between global macroscale simulations—using properties obtained from those microscale solutions—and corresponding fully resolved simulations. In this work, we not only compute effective material tensors by numerically solving microscale problems, but also compare the obtained effective continuum description with fully resolved direct numerical simulation (DNS) of the fluid flow inside and above the poroelastic surface. In this way, we can assess the accuracy of the effective model quantitatively both in the interior and near the interface of the medium with a free-flowing fluid.

A particularly important contribution is the insight it provides on how the velocity and the stress interface conditions between the poroelastic medium and the free fluid region model the microscale effects in an averaged manner. Gopinath & Mahadevan [28] as well as Minale [33] point out that the effective interface condition for a poroelastic region may in fact be more straightforward to match, compared to the rigid porous material, since there is a natural way to balance the fluid stress from the free fluid with the solid stress of the surface material. However, there exists no validation—where the interface boundary conditions for displacement, fluid velocity and pressure are coupled to the Stokes equations above—for a non-trivial flow, where there is transport across the interface. Most works treating the boundary conditions are empirical [34–36]. Those contributions which have recently treated the interface problems from first principles have focused on rigid porous media and one-dimensional problems, such as the laminar channel flow [37,38] only (for which there is no transfer of mass or momentum between the material and the free fluid), or infiltration flow only [39].

In summary, the objectives of the current work are to (i) present a framework, derived using multi-scale expansion, suitable to model flow through and over poroelastic materials, (ii) validate the framework with respect to the fully resolved direct numerical simulations, and (iii) gain insight on stress transfer near the interface and evaluate the accuracy of interface boundary conditions. Our work is partially the numerical counterpart of the analytical and asymptotic study by Gopinath & Mahadevan [28]; we aim at computing (instead of physically modelling) physical material properties of anisotropic poroelastic materials as well as the interface with the free flow. While Gopinath & Mahadevan [28] consider particularly biologically relevant microstructures of ordered or disordered filaments, we consider connected materials consisting of linked spheres and ellipsoids.

This paper is organized as follows. In §2, we introduce both the microscale and the macroscale/effective equations governing our problem, which consists of a poroelastic material at the interface with a moving fluid. A method to compute the effective properties of poroelastic materials is presented in §3. In the same section, we provide effective tensor results for cubic-symmetric and monoclinic-symmetric poroelastic materials and analyse them. A lid-driven cavity problem to investigate poroelastic material response to a steady two-dimensional flow vortex is proposed and solved using the homogenized equations in §4. In the same section, we report results obtained from resolved direct numerical simulations and explain the shear stress transfer between free fluid and poroelastic medium. In §5, we discuss the limitations of the presented theory. Finally, in §6, we conclude this work and outline future directions.

## 2. Micro- and macroscale equations describing a poroelastic bed

**Figure 1.**
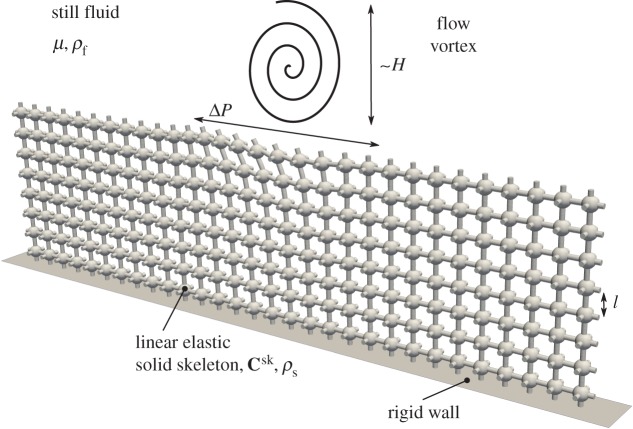
Illustration of a free fluid vortex interaction with a poroelastic material. The material is composed of multiple instances of unit-cell skeleton geometry—sphere with circular cylinder connections in all directions. We represent a slice one pore structure thick. The unit cell is a cube with side length *l*. The solid skeleton is characterized by linear elasticity tensor **C**^sk^ and density *ρ*_*s*_. The flow vortex has a length scale *H* and causes a characteristic pressure difference Δ*P*. The solid skeleton is deformed under the influence of the free fluid vortex.

For a dense poroelastic medium exposed to a free flow (figure 1), one may define at least two length scales; a microscopic (pore) scale *l*, which characterizes the size of voids in the material and a macroscopic (global) scale *H*, which characterizes the size of the large-scale processes in the medium or nearby. The large-scale flow is characterized also by the created pressure difference Δ*P*. In this section, we present the microscale equations that resolve every scale in the full physical domain, and a set of macroscale equations that model the effective average behaviour of the poroelastic bed under a given set of assumptions. Although the effective field equations for describing a fluid-saturated poroelastic material are known [6,28], we re-derive them from first principles in the electronic supplementary material, appendices. The main reason is to uncover the detailed microscale problems in unit cells that are required for determining the physical coefficients appearing in the macroscale systems.

### (a) Microscale governing equations

The microscale equations resolving the fluid-structure physics at scale ∼*l* are the conservation of mass and momentum. For a Newtonian fluid with constant density *ρ*_*f*_ and viscosity *μ*, the momentum in the free fluid region and within the porous skeleton is governed by the incompressible Navier–Stokes equations, see [40], p. 147, which are

2.1 $$\begin{array}{rl} \rho_{f}[\partial_{t}\text{u}+(\text{u}\cdot \nabla )\text{u}] & =-\nabla p+\mu \Delta \text{u}, \end{array}$$

2.2 $$\begin{array}{rl} \nabla \cdot \text{u} & =0, \end{array}$$

where **u** and *p* are velocity and pressure fields, respectively.

The material is defined by the solid skeleton density *ρ*_s_ and linear elasticity tensor **C**^sk^. Assuming an isotropic material the elasticity tensor is defined by Young's modulus *E* and Poisson's ratio *ν*. The solid skeleton momentum is governed by a balance between solid inertia and stress, obtained using the linear stress–strain relationship

2.3 $$\rho_{s}\partial_{t}^{2}\text{v}=\nabla \cdot \left\{{\text{C}}^{\mathrm{sk}}:\frac{1}{2}\left[\nabla \text{v}+{\left(\nabla \text{v}\right)}^{T}\right]\right\},$$

where **v** is the displacement field of the solid skeleton (see [41], eqn 3.38). To couple the fluid and structure problems, the no-slip condition and continuity of stress is prescribed at the boundary between solid skeleton and surrounding fluid, i.e.

2.4 $$\text{u}=\partial_{t}\text{v}\;\text{and}\;\left\{-p\delta +\mu \left[\nabla \text{u}+{\left(\nabla \text{u}\right)}^{T}\right]\right\}\cdot \hat{n}=\left\{{\text{C}}^{\mathrm{sk}}:\frac{1}{2}\left[\nabla \text{v}+{\left(\nabla \text{v}\right)}^{T}\right]\right\}\cdot \hat{n}.$$

Here, ***δ*** is the second-rank identity tensor and $\hat{n}$ is unit-normal vector at the boundary. Solving the governing equations everywhere at the pore scale is computationally very expensive due to a globally large domain and due to requirement of fine resolution near the pores. This motivates the development of an alternative continuum description, where the pore fluid and solid are considered as one composite, which given appropriate equations and constitutive relations presents the average behaviour of the actual poroelastic bed.

### (b) Effective field equations

**Figure 2.**
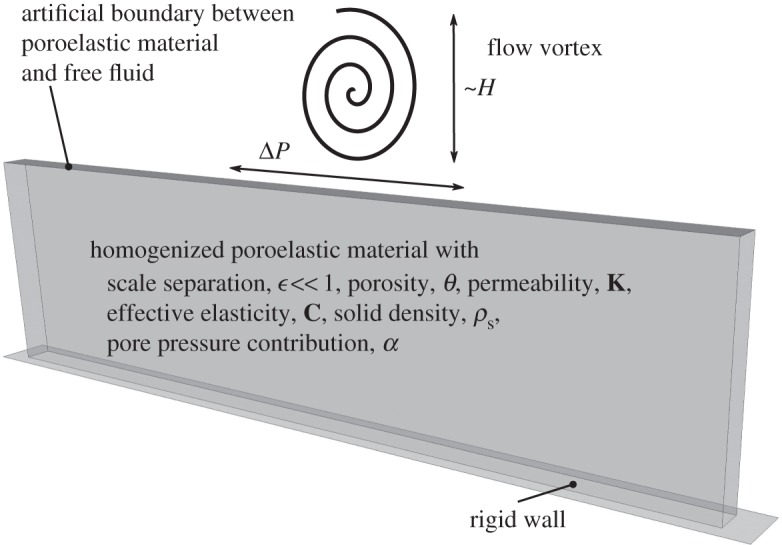
Illustration of a free fluid vortex interaction with a poroelastic material. The material is described in a homogenized two-domain setting and is characterized by the scale separation parameter *ϵ* and porosity *θ*, as well as effective permeability **K**, effective elasticity **C**, density *ρ*_s_ and pore pressure contribution ***α***. The homogenized domain is not visibly deformed under the influence of free fluid vortex. There is an artificial boundary introduced between the homogenized poroelastic material and free fluid.

We divide the physical domain into two parts: one containing only the free fluid, and the other containing the fluid and solid skeleton (figure 2). The free-fluid region is governed by the Navier–Stokes equations (2.1) and (2.2).

The continuum description of the composite (solid and fluid) is based on a separation between the pore-scale and the system scale. Mathematically, this can be formulated by a scale separation parameter *ϵ*=*l*/*H*≪1. The effective displacement field **v** of the homogenized poroelastic material (figure 2) is governed by

2.5 $$(1-\theta )\rho_{s}\partial_{t}^{2}\text{v}=\nabla \cdot \left[\text{C}:\frac{1}{2}\left(\nabla \text{v}+{\left(\nabla \text{v}\right)}^{T}\right)-\alpha p\right].$$

Here, *θ*=*V*_*f*_/*V* is the porosity (which in general is a function of space, but in this work is a constant), where *V*_*f*_ is the fluid phase volume and *V* is the total volume of the composite in one unit cell (defined later). Moreover, **C** is the fourth-rank effective elasticity tensor of the material and ***α*** is the coefficient for the contribution of the pore pressure (*p*) in the total stress. Whereas the microscale elasticity **C**^sk^ only depends on *E* and *ν*, the effective tensor **C** in general depends on the porosity, pore geometry (as modelled in the current work) and on the particular type of boundary condition imposed near the interface with the free fluid, or the impermeable walls [28]. The tensor ***α*** does not have a correspondence in the microscale and is an effect of solid skeleton deformation due to seepage flow through the pores. We characterize in detail both **C** and ***α*** for two different poroelastic media in §3.

Moreover, for sufficiently dense poroelastic material the fluid flow between the pores is slow, such that inertial effects are negligible. Therefore, the pore pressure is the dominant contribution in the fluid flow and the leading-order equation is the (relative) Darcy's Law,

2.6 $$\text{u}-\theta \partial_{t}\text{v}=-\frac{\text{K}}{\mu }\cdot \nabla p.$$

This expression relates the gradient of the pore pressure, the solid velocity ∂_*t*_**v** and the flow field **u** in the poroelastic medium to each other. Here, **K** is the interior permeability tensor. The term *θ*∂_*t*_**v** arises due to viscous friction between the solid structure and the pore fluid; if there is a motion of the solid skeleton, the surrounding pore fluid is dragged along through boundary condition (2.4).

Finally, the conservation of mass requires that

2.7 $$\nabla \cdot \text{u}=\text{D}:\frac{1}{2}\partial_{t}\left(\nabla \text{v}+{\left(\nabla \text{v}\right)}^{T}\right)-E\partial_{t}p,$$

where **D**=*θ****δ***−***α*** is a dimensionless second-rank tensor. It determines how the strain of the displacement (caused either by the flow through the pores or by a boundary condition) modifies the solid structure volume within one pore, thus squeezing the pore fluid in or out of the pore. The scalar E characterizes the change of solid structure volume within one pore with respect to time-varying pressure, which, similarly to solid strain, can cause change in pore fluid content and consequently introduce apparent compressibility of the flow field.

The system of equations (2.5)–(2.7) determines the seven unknowns (**u**,**v** and *p*), but by combining (2.6) with (2.7), the system can be reduced to four unknowns (**v** and *p*). The fluid velocity **u** can then be computed as a post-processing step once pressure and displacement fields are known. Following this approach, the equation for the pore pressure is

2.8 $$E\partial_{t}p-\nabla \cdot \left(\frac{\text{K}}{\mu }\cdot \nabla p\right)=-\alpha :\frac{1}{2}\partial_{t}\left(\nabla \text{v}+{\left(\nabla \text{v}\right)}^{T}\right).$$

Note that the effective system above for describing a poroelastic system can be derived using multi-scale expansion (see §3 and appendices in electronic supplementary material), motivated using mixture theory or physically modelled. The major challenge is to use this developed effective system with appropriate boundary conditions at interfaces with solid walls, free fluids or other structures to describe problems arising from various applications. The aim of this work is to provide a framework from which one can form a fully closed effective system to describe the response and interaction of a poroelastic material with a surrounding free fluid. In the next section, we therefore provide the needed boundary conditions between a poroelastic material and a rigid wall, and also a free fluid.

### (c) Effective interface conditions

#### (i) Conditions for the poroelastic bed

To solve the governing equations for the poroelastic material, one needs to impose boundary conditions for both the pore-pressure (2.8) and the displacement field (2.5) equations. On rigid walls, we impose (similar to [28]) zero displacement and zero transpiration (normal fluid velocity), which through the relative Darcy's equation (2.6) leads to

2.9 $$\text{v}{|}_{\mathrm{rw}}=0\;\text{and}\;{\frac{\partial p}{\partial \hat{n}}|}_{\mathrm{rw}}=0.$$

Here, ‘*rw*’ means ‘rigid wall’ and $\hat{n}$ is the unit-normal vector at the wall. Physically, the no-slip condition should be satisfied at the wall, but this is not compatible with the leading-order presentation of a poroelastic media based on Darcy's Law. Darcy's Law only describes the direct proportionality between the pore-pressure gradient and the velocity, and does not include any macroscopic diffusion effects.

At the artificial interface with the free fluid, shown in figure 2, we impose a pressure continuity condition

2.10 $$p^{-}=p.$$

Here, the pore pressure below the interface is denoted by *p*^−^ and pressure of the free fluid above the interface with *p*. Note that the choice of condition for pressure depends on the assumptions made about the flow as well as the material geometry. For example, Lācis & Bagheri [42] have shown that, if the interface velocity caused by the shear stress is of the same order as the velocity induced by the pore pressure gradient, the pressure continuity is the leading-order boundary condition for any pore geometry. On the other hand, Mikelić and colleagues [43,44] have shown that, if velocity contribution from the shear stress at the interface is one order higher than contribution from the pressure gradient, there is a pressure jump for anisotropic pore geometry. However, if the pore geometry is isotropic/cubic-symmetric, the pressure continuity is still applicable. Finally, for the material displacement, we impose continuity of stresses at the interface

2.11 $$\left[\text{C}:\frac{1}{2}\left(\nabla \text{v}+{\left(\nabla \text{v}\right)}^{T}\right)-\alpha p\right]\cdot \hat{n}=\left[-p\delta +\mu \left(\nabla \text{u}+{\left(\nabla \text{u}\right)}^{T}\right)\right]\cdot \hat{n}.$$

We thus assume, similarly to [28], that the total stress of the free fluid is transferred to the total effective stress of the interior poroelastic medium. In general, the effective elasticity of the composite near the interface could however be different from its value in the interior (it is argued in general to depend on boundary conditions [28]) and, to arrive to a more accurate boundary condition, one could construct an interface cell with an elasticity problem, similarly as done for the velocity boundary condition [42]. One objective of this paper is to understand how the fluid shear stress is transferred to the solid stress (first across the interface then inside the bed) and if the interface correction is necessary; in §4, we will show that approximation of the interface effective stress with the interior parameters is able to capture the transfer of stress reasonably well.

#### (ii) Conditions for the free fluid

To solve for the Navier–Stokes equations in the free fluid domain, we need to impose velocity—in principle, one could also have stress condition, but it is already used for the displacements of the poroelastic material—boundary conditions at the interface. In this work, we extend the velocity boundary condition derived by Lācis & Bagheri [42] for a rigid porous bed to include poroelasticity.^1^ The condition for the tangential interface velocity is

2.12 $$\text{u}\cdot \hat{\tau }=\partial_{t}\text{v}\cdot \hat{\tau }+\left(-\frac{{\text{K}}^{\mathrm{if}}}{\mu }\cdot \nabla p^{-}+\text{L}:\left[\nabla \text{u}+{\left(\nabla \text{u}\right)}^{T}\right]\right)\cdot \hat{\tau },$$

where the unit vector $\hat{\tau }$ denotes both tangential directions of the surface. Note that the pressure gradient is the pore-pressure gradient from poroelastic material side of the interface (hence the minus superscript), whereas the flow velocity field **u** is on the free-fluid side. The interface velocity has two distinct contributions: (i) the no-slip contribution governed by the movement of the solid structure; (ii) the slip contribution, which is caused by the porosity of the solid structure and depends both on pore pressure gradient and free-fluid shear. The slip contribution is characterized by the second-rank interface permeability tensor **K**^*if*^ and the third-rank slip length tensor **L**. For a dense material, the first slip term scales as *l*^2^ and is significantly smaller than the second term, which scales as *l*. The motion of the poroelastic material or the no-slip contribution depends not only on the pore-length *l*, but also on the elasticity of the material and the flow regime. The condition for the normal ‘penetration’ interface velocity component is set by mass conservation, i.e.

2.13 $$\text{u}\cdot \hat{n}=\partial_{t}\text{v}\cdot \hat{n}-\left(\frac{\text{K}}{\mu }\cdot \nabla p^{-}\right)\cdot \hat{n},$$

where **K** is the interior permeability tensor defined in equation (2.6) of the porous medium and ∇*p*^−^ denotes the pressure gradient when approaching the interface from the bed. Similarly as for the tangential component, the velocity has two parts—the no-slip part and the ‘slip’ part. The slip in the normal direction is essentially the fluid mass transport in and out of the poroelastic material, which has to be equal to the relative velocity from the interior. This same condition also arises from the interface cell [42], because the tensor **L** components corresponding to penetration interface velocity are zero.

In the next section, we will compute the physical parameters which characterize the interior poroelastic medium (**C**, ***α***, **K** and E) as well as the parameters characterizing the interface with the free fluid (**K**^*if*^,**L**) for two specific pore-scale geometries. In §4, we will investigate in more details interface conditions at boundary between poroelastic material and free fluid by comparing the effective model against the microscopically resolved system using the lid-driven cavity problem.

## 3. Properties of poroelastic material exposed to free fluid

**Figure 3.**
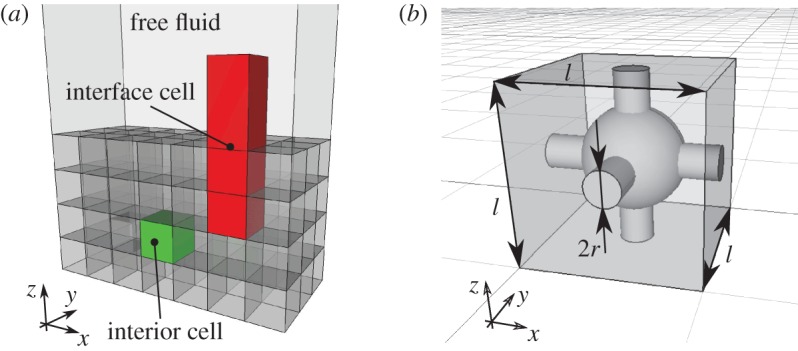
In (*a*), we show a constructed poroelastic material consisting of 84 unit cells with volume *l*^3^, which can exhibit only cubic-symmetry due to boundaries between the unit cells. Above thematerial, there is a free fluid. Interior unit cell is depicted with light grey (green online), interface cell is depicted with dark grey (red online). In (*b*), we show a cubic-symmetric micro-structure placed in a single interior unit cell. The structure is built using a sphere with radius *R*=0.3*l* and cross-connected cylinders with radius *r*=0.1*l*. (Online version in colour.)

We now turn to determining the effective properties of a poroelastic material exposed to the free fluid, by introducing and solving a set of particular microscale problems in an interior unit cell and in an interface cell. We focus on a periodic solid structure that can be obtained by duplicating a single pore structure in all directions. The sketch in figure 3*a* shows how the material and its interface with free-fluid is divided into cubic interior cells and elongated rectangular interface cells. The effective parameters are computed by solving two elasticity problems (to obtain **C**, ***α*** and E) and one fluid problem (to obtain **K**) in the interior cell and two fluid problems in the interface cell (to obtain **K**^*if*^,**L**). These microscale problems are first illustrated for the interior domain using a weakly and a strongly anisotropic microstructure, before we move on to the microscale problems at the interface with the free fluid.

### (a) Interior of the poroelastic material

#### (i) Microscale problems for elasticity parameters

The effective poroelastic bed obtained from the unit-cell approach cannot have fully isotropic elastic properties due to the boundaries between cubic unit cells, as shown in figure 3*a*. The resulting effective elasticity **C** will at most exhibit a cubic symmetry; a symmetry, which is defined by planes—parallel to the unit-cell sides and diagonal across the unit cell in all directions—going through the centre of the cube.

In order to preserve cubic symmetry, the unit cell can be filled with any structure that itself is cubic symmetric, such as Wigner–Seitz grains [32,13] or cube with spherical holes [45]. In this work, we use a sphere at the centre of unit cell with radius *R*, which is connected to neighbouring cells via circular cylinders of radius *r*, as shown in figure 3*b*. The solid skeleton structure is built from isotropic elastic material with elasticity tensor

$$C_{ijkl}^{\mathrm{sk}}=E\left\{\frac{\nu }{\left(1+\nu )(1-2\nu \right)}\delta_{ij}\delta_{kl}+\frac{1}{2(1+\nu )}(\delta_{ik}\delta_{jl}+\delta_{il}\delta_{jk})\right\}.$$

The effective elasticity tensor can be determined (see the electronic supplementary material, appendix B, for derivation) using a third-rank displacement test tensor ***χ*** as

3.1 $$\text{C}=(1-\theta ){\text{C}}^{\mathrm{sk}}+{\text{C}}^{\mathrm{sk}}:\left\langle \frac{1}{2}\left[\nabla \chi +{\left(\nabla \chi \right)}^{T}\right]\right\rangle ,$$

where we define transpose of the fourth-rank tensor **∇*χ*** acting on the first two indices (**∇*χ***)^*T*^_*ijkl*_=(**∇*χ***)_*jikl*_. The expression above provides a direct linear link between the micro- and macroscale elasticity tensors through the known porosity *θ* and the pore-scale geometry captured by ***χ*** that will be computed by solving equations (3.3) and (3.4). Here, it is assumed that the skeleton elasticity tensor **C**^sk^ is constant in space. The brackets denote the volume average over the interior unit-cell volume, which is filled either by solid or by fluid

3.2 $$\langle \;f\rangle =\frac{1}{l^{3}}\int_{V_{\sigma }}f\;dV,$$

where *V*_*σ*_ is the volume of either the solid or fluid phase. In general, the effective properties should be re-evaluated as the porosity changes. However, we assume small displacements of the effective system so that the porosity is roughly constant and, therefore, it is enough to compute the averages only once.

The microscale displacement tensor ***χ*** is defined in the solid phase (having volume *V*_s_) of the unit cell and, see [13] and the electronic supplementary material, appendix B, is the solution of the following problem:

3.3 $$\begin{array}{rl} \nabla \cdot \left[{\text{C}}^{\mathrm{sk}}:\frac{1}{2}\left\{\nabla \chi +{\left(\nabla \chi \right)}^{T}\right\}\right] & =0, \end{array}$$

3.4 $$\begin{array}{rl} \left[{\text{C}}^{\mathrm{sk}}:\frac{1}{2}\left\{\nabla \chi +{\left(\nabla \chi \right)}^{T}\right\}\right] & \cdot \hat{n}=\left[{\text{C}}^{\mathrm{sk}}:\delta^{\left(4\right)}\right]\cdot \hat{n}. \end{array}$$

Here, the fourth-rank identity tensor appearing in the boundary condition is defined as ${\left(\delta^{\left(4\right)}\right)}_{ijkl}=\delta_{ik}\delta_{jl}$. This equation corresponds to a standard steady linear-elasticity problem generalized to third-rank tensor subjected to different combinations of surface loading (applied on the interface between the solid skeleton and the pore fluid) in order to characterize the response of the structure to all possible surface loading scenarios. To complete the formulation, periodic boundary conditions are applied to solid surfaces, which are in contact with the boundary of the unit cell. In order to render the solution unique, we impose constraints on average values of displacement [13] using penalty terms in weak formulation [46].

The unit-cell domain has been meshed^2^ using GMSH software [47] and equations (3.3) and (3.4) has been solved using FreeFEM++ [48]. The sphere radius is *R*=0.3*l* and the cylinder radius is *r*=0.1*l*, which results in porosity *θ*=0.85. Assuming Poisson's ratio to be *ν*=0.3 (due to the linearity of the problem, the solution is valid for any Young's modulus value *E*), the effective elasticity tensor for the cubic-symmetric poroelastic medium in Voigt notation [41], p. 136 is

3.5 $$\text{C}=\left(\begin{array}{cccccc} 4.792 & 0.239 & 0.239 & 0 & 0 & 0\\ 0.239 & 4.794 & 0.239 & 0 & 0 & 0\\ 0.239 & 0.239 & 4.792 & 0 & 0 & 0\\ 0 & 0 & 0 & 0.246 & 0 & 0\\ 0 & 0 & 0 & 0 & 0.246 & 0\\ 0 & 0 & 0 & 0 & 0 & 0.246 \end{array}\right)\cdot 10^{-2}E.$$

Note that the entries of the effective elasticity tensor are significantly smaller (up to 300 times) compared with these of the skeleton elasticity. This is because most of the solid mass has been removed, leaving only 15% solid volume fraction if compared to a completely filled case, hence the resulting material is much softer. Another observation is that the shear coefficients (diagonal elements in 3×3 bottom right matrix block) are now much smaller compared with pressure-wave coefficients [49], p. 22 (diagonal elements in 3×3 top-left matrix block). This effect can be attributed to connecting net of cylinders between spheres; the cylinders are much easier to bend, compared to bulk material, i.e. there is no continuous support from the sides, as there would be in the continuous material case. The effective tensor can be characterized by three independent parameters^3^ and has the form of a material with cubic symmetry [41], p. 99.

The other two elasticity-related effective parameters ***α*** and E can be computed through expressions^4^ involving the inverse of the fourth-rank elasticity tensor effective elasticity **C** [28], eqns 2.4,2.7,2.8. Alternatively, one can solve for an additional test displacement field ***η*** governed by the linear elasticity problem

3.6 $$\begin{array}{rl} \nabla \cdot \left[\frac{{\text{C}}^{\mathrm{sk}}}{E}:\frac{1}{2}\left\{\nabla \eta +{\left(\nabla \eta \right)}^{T}\right\}\right] & =0, \end{array}$$

3.7 $$\begin{array}{rl} \left[\frac{{\text{C}}^{\mathrm{sk}}}{E}:\frac{1}{2}\left\{\nabla \eta +{\left(\nabla \eta \right)}^{T}\right\}\right] & \cdot \hat{n}=\delta \cdot \hat{n}. \end{array}$$

Note that this problem is driven by different surface forcing compared to the ***χ*** problem, but is otherwise solved under the same conditions. Given ***η***, the pore-pressure contribution tensor is computed from

3.8 $$\alpha =\theta \delta +\left\langle \frac{{\text{C}}^{\mathrm{sk}}}{E}:\frac{1}{2}\left[\nabla \eta +{\left(\nabla \eta \right)}^{T}\right]\right\rangle ,$$

and the coefficient describing the poroelastic material response to the time-variation of the pore pressure is computed from

$$E=\left\langle \frac{\nabla \cdot \eta }{E}\right\rangle .$$

By solving (3.6) and (3.7) for the particular microstructure with cubic symmetry shown in figure 3*b*, we obtain

$$\alpha =0.9789\;\delta \;\mathrm{and}\;E=0.1571\;E^{-1}.$$

The tensor ***α*** is not unity, therefore one can conclude that the solid phase of the poroelastic material is not incompressible. This conclusion agrees with the used elasticity parameter (we use *ν*=0.3, while incompressible solids have *ν*=0.5). However, it is non-trivial to use tensor ***α*** as a measure of compressibility, because it is also a function of porosity *θ*.

#### (ii) Microscale problem for permeability tensor

Turning attention to the fluid flow, the poroelastic material is characterized by a permeability [5], which in our framework is obtained by solving the following Stokes problem in the interior unit cell (figure 3*b*):

3.9 $$\begin{array}{rl} -\nabla A+\nabla^{2}K & =-\delta , \end{array}$$

3.10 $$\begin{array}{rl} \nabla \cdot K & =\text{0}. \end{array}$$

Here, K is a second-rank tensor field; $K_{ij}$ is the *i*th velocity component of *j*th vectorial Stokes problem associated with the pressure field $A_{j}$. The three vectorial Stokes problems characterize how the pore flow responds to volume forcing in one spatial direction at a time. This equation system is complemented with no-slip boundary condition at the interface with the solid skeleton, and periodic boundary conditions at the sides of the unit cell. For uniqueness, we require that the average pressure field is zero. The effective permeability tensor for the cubic symmetric material is then obtained by averaging the field K over the fluid volume in the unit cell as

3.11 $$\text{K}=\langle K\rangle =2.32\cdot 10^{-2}\;l^{2}\;\delta .$$

We observe that the permeability tensor is characterized by a single constant value, which is characteristic for isotropic flow in the poroelastic medium. Hence, the cubic-symmetry of the poroelastic material is not visible in permeability tensor.

#### (iii) Effects of significant anisotropy at the pore-scale

**Figure 4.**
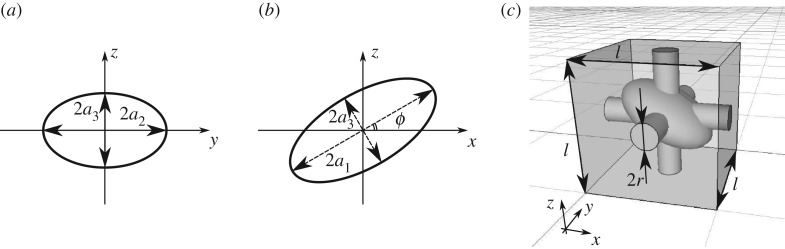
Definition of an ellipsoid in the centre of representative volume element in poroelastic medium. Panel (*a*) shows the ellipsoid slice in (*y*,*z*)-plane. This particular ellipsoid is aligned with axis. Panel (*b*) shows the tilt of the ellipsoid around *y*-axis with angle *ϕ*. Panel (*c*) shows the micro-structure geometry with one plane of symmetry (monoclinic symmetric material). The ellipsoid parameters are *a*_1_=0.4*l*, *a*_2_=0.3*l*, *a*_3_=0.2*l* and turn angle is *ϕ*=30°. Cylinder radius is *r*=0.1*l*.

With the aim of providing a framework that can cope with any periodic microstructure, we compute the effective parameters for a strongly anisotropic microstructure, namely, a tilted ellipsoid at the centre of the unit cell, as shown in figure 4. Note that the cubic symmetry in the poroelastic material is broken by this structure, since it has less symmetry planes compared with the cubic unit cell. The ellipsoid semi-axes have lengths *a*_1_, *a*_2_ and *a*_3_ in *x*-, *y*- and *z*-directions, respectively. The ellipsoid can be rotated around the *y*-axis by an angle *ϕ* as shown in figure 4*b*. In order to assess how anisotropy at the pore-scale modifies the effective parameters at the macroscale, we choose parameters that break as many symmetries as possible; *a*_1_=0.4*l*, *a*_2_=0.3*l* and *a*_3_=0.2*l* and *ϕ*=30°. This results in a porosity *θ*=0.86 and structure with only one symmetry plane.

Using expression (3.1), we evaluate the effective elasticity tensor for the titled ellipse structure by solving the microscale displacement problem for ***χ*** (3.3) and (3.4),

3.12 $$\text{C}=\left(\begin{array}{cccccc} 4.668 & 0.283 & 0.233 & 0 & 0.139 & 0\\ 0.283 & 4.638 & 0.209 & 0 & -0.002 & 0\\ 0.233 & 0.209 & 4.031 & 0 & 0.084 & 0\\ 0 & 0 & 0 & 0.168 & 0 & -0.010\\ 0.139 & -0.002 & 0.084 & 0 & 0.205 & 0\\ 0 & 0 & 0 & -0.010 & 0 & 0.258 \end{array}\right)\cdot 10^{-2}E.$$

The form of **C** corresponds to a monoclinic material symmetry [41], p. 96, and nearly all the elements of the tensor differ from each other, as expected due to the anisotropic pore structure. This symmetry property is invariant with respect to transformation of coordinate axes; by using a different coordinate system, one would only be able to reposition the zero entries in the elasticity tensor to different rows or columns. Moreover, the magnitude of the elements are of the same order as for the cubic-symmetric geometry, since the two cases have nearly the same porosity.

By solving for the displacement field ***η*** problem (3.6) and (3.7), we obtain via expression (3.8)

$$\alpha =\left(\begin{array}{ccc} 0.9793 & 0 & -0.0009\\ 0 & 0.9795 & 0\\ -0.0009 & 0 & 0.9821 \end{array}\right),$$

where we observe that the tensor cannot be characterized by a single constant as for the cubic-symmetric material, but by four distinct coefficients. The tensor ***α*** can be understood as a measure of volume force in the poroelastic medium caused by pore-pressure gradient via seepage flow. The diagonal terms are different because of the different lengths of the ellipsoid semi-axes *a*_1_, *a*_2_ and *a*_3_. Owing to this difference, the area of the solid structure (figure 4*c*), exposed to flow in *x*-, *y*- and *z*-directions, is different. Consequently, it results in a different drag force for the same pore-pressure gradient in different directions. The off-diagonal term is, on the other hand, caused by the tilt of the ellipsoid, which gives raise to non-zero pressure force, when projected in *x*-direction, due to flow in *z*-direction and vice versa. The elastic response to time variation of pressure is

$$E=0.1465\;E^{-1}.$$

The slightly smaller value for the ellipsoid (compared to the sphere) can be attributed to the shape of the pore structure at the centre of the unit cell. The ellipsoid is thinner in one direction compared to sphere, which leads to larger strain from the same displacement values and consequently a body which is less compressible.

By solving the fluid problem (3.9) and (3.10) at the pore-scale for the monoclinic symmetric geometry, we obtain effective permeability tensor

3.13 $$\text{K}=\left(\begin{array}{ccc} 2.51 & 0 & -0.10\\ 0 & 2.25 & 0\\ -0.10 & 0 & 2.22 \end{array}\right)\cdot 10^{-2}\;l^{2}.$$

The second-rank permeability tensor has a similar features as the pore-pressure contribution tensor ***α***. It has four independent parameters, which are again set by four geometric parameters—semi-axes lengths *a*_1_, *a*_2_ and *a*_3_ as well as turn angle *ϕ*. The off-diagonal term shows that a tilted ellipse generates flow in the *x*-direction if exposed to pressure a gradient along *z*, and vice versa. This effect is the same as for a tilted plate exposed to an incoming parallel free stream. Owing to the tilt, a tangential flow with respect to the incoming free stream appears.

### (b) Microscale problems for interface conditions

In order to finalize the homogenized model, one needs effective tensors for the velocity boundary conditions of the free fluid in contact with the poroelastic material. We can determine the velocity boundary conditions (the interface permeability **K**^if^ and the Navier-slip tensor **L**) by solving a set of microscale problems in an interface cell (figure 3*a*) as shown for rigid porous media by Lācis & Bagheri [42].

To derive the microscale problems in the interface unit cell, we decompose the flow above the interface into a fast flow **U** and a perturbation **u**^+^ flow

3.14 $$\text{u}=\text{U}+{\text{u}}^{+}.$$

**Figure 5.**
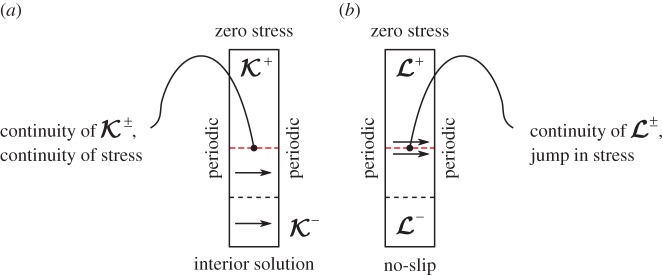
A two-dimensional illustration of the interface-cell problem for $K_{ij}$ (*a*) and $L_{ijk}$ (*b*). The interface cell has volume forcing in the *x*-direction below the interface (uppermost dashed line) for the $K_{i1}$ problem (*a*). The interface cell has a jump in stress at the interface location, which results in a force along *x* for the $L_{i13}$ problem (*b*). The sides of the interface cell are exposed to periodic boundary conditions, and the top—to zero-stress condition.At the bottom of the cell either the interior solution (for the $K_{ij}$ problem) or no-slip (for the $L_{ijk}$ problem) is prescribed. The dashed lines correspond to boundaries of unit cells. Above the last unit cell, there is only free-fluid. (Online version in colour.)

Below the interface, there is only slow flow **u**^−^. The flow perturbation velocity is the cause of the slip velocity at the interface with porous or poroelastic material [42]. Note that the global pressure difference Δ*P* is driving the fast flow **U** above the interface as well as the slow flow **u**^−^ below the interface, whereas **u**^+^ is driven by the processes in the poroelastic material. In order to determine the perturbation velocity, one can arrive to a solution in the interface cell (figure 3*a*) using an assumption of linear superposition

$${\text{u}}^{\pm }=\partial_{t}\text{v}-\frac{K^{\pm }}{\mu }\cdot \nabla p^{-}+L^{\pm }:\left[\nabla \text{u}+{\left(\nabla \text{u}\right)}^{T}\right],$$

in which the unknown tensorial fields $K^{\pm }$ and $L^{\pm }$ are velocity response fields to test forcing, which corresponds to pressure gradient (volume forcing) and surface stress (surface forcing). Fields $K^{-}$ and $L^{-}$ are correspondingly defined below the interface (illustrated using the dash red line), and fields $K^{+}$ and $L^{+}$ are defined above the interface, as sketched in figure 5. In the next sections, we introduce problems for all of the introduced test fields and solve them for both microscale geometries.

#### (iv) Interface permeability tensor **K**^if^

The interface permeability second-rank tensor field below the interface $K^{-}$ is governed by

3.15 $$-\nabla A^{-}+\nabla^{2}K^{-}=-\delta .$$

The boundary conditions at the sides of this domain are periodic (same as for interior cell), however, at the bottom of the domain one has to use the interior solution provided by the interior problem (3.9) and (3.10), see figure 5*a*. At the interface, the continuity of permeability fields

3.16 $$K^{-}=K^{+}$$

is employed. Since the permeability field in the upper part of the interface cell is also unknown, it can be found using an unforced Stokes momentum equation:

3.17 $$-\nabla A^{+}+\nabla^{2}K^{+}=0.$$

The volume force in Stokes equations above the interface does not exist, because the global driving pressure above the interface acts on the fast flow, not on the perturbation (3.14). To complete the two-domain formulation of the interface cell, incompressibility constraints are added, no-slip condition is imposed at the surface of solid skeleton, periodic boundary conditions are applied at the sides, zero stress condition at the top of the cell and stress continuity at the interface,

3.18 $$\left\{-\delta A^{-}+\left[\nabla K^{-}+{\left(\nabla K^{-}\right)}^{T}\right]\right\}\cdot \hat{n}=\left\{-\delta A^{+}+\left[\nabla K^{+}+{\left(\nabla K^{+}\right)}^{T}\right]\right\}\cdot \hat{n},$$

as shown in figure 5*a*. One can observe from equations (3.15)–(3.18) that the outlined problem is a combination of Stokes problems exposed to unit volume-forcing in all possible directions below the interface. For example, the first column of the tensor $K^{\pm }$ field ($K_{i1}^{\pm }$) corresponds to flow response to unit volume forcing in the *x*-direction, as shown in figure 5*a*. Thus, this test problem is used to characterize the pore-pressure gradient (which is a volume force) contribution to velocity near the interface. Finally, after solving the coupled two-domain Stokes problem, one can obtain the effective interface permeability, by employing volume average ${\text{K}}^{\mathrm{if}}=\langle K^{+}\rangle$ in *l*^3^ cube above the interface.

Evaluating^5^ the interface permeability tensor gives

3.19 $${\text{K}}^{\mathrm{if}}=\left(\begin{array}{ccc} 3.20 & 0 & 0\\ 0 & 3.27 & 0\\ 0 & 0 & 2.33 \end{array}\right)\cdot 10^{-2}l^{2}\;\text{and}\;{\text{K}}^{\mathrm{if}}=\left(\begin{array}{ccc} 3.42 & 0 & 0\\ 0 & 3.21 & 0\\ -0.10 & 0 & 2.23 \end{array}\right)\cdot 10^{-2}l^{2},$$

for cubic-symmetric and monoclinic-symmetric pore-scale geometries, respectively. One can observe that the interface-normal permeability components (for velocity component *u*_*z*_, last row of the matrix) are the same as interior ones (set by conservation of mass [42]), but tangential components are different. The interface permeability for the tangential components is larger than the interior one, which can be explained by the fact that there is no friction from the upper part of the interface cell, while the interior cell would be exposed to friction from neighbouring cells. In addition, the interface permeability matrix for monoclinic symmetric pore geometry is no more symmetric, that is the effect of anisotropy has vanished for *u*_*x*_ velocity component at the interface. The reason for this coefficient vanishing could be the fact that the anisotropy is too weak to overcome dissipation at the cylinder on the top of the last micro-structure and does not contribute for the interface velocity.

#### (v) Interface Navier-slip tensor **L**

When there is a flow over a porous or poroelastic material, there is a slip velocity induced proportional to free fluid shear stress, as theoretically derived by Mikelić & Jäger [37] for a one-dimensional channel flow and by Lācis & Bagheri [42] for a general three-dimensional set-up.

The slip length problem, unlike the permeability problem, is specific to the interface, i.e. there exists no analogous problem for the interior domain. The slip third-rank tensor fields are also governed by Stokes equations

3.20 $$\begin{array}{rl} -\nabla B^{-}+\nabla^{2}L^{-} & =0, \end{array}$$

3.21 $$\begin{array}{rl} -\nabla B^{+}+\nabla^{2}L^{+} & =0, \end{array}$$

below and above the interface, respectively, see figure 5*b*. As before, the fields in two domains are connected through continuity condition $L^{-}=L^{+}$. Momentum equations in this test problem are not exposed to volume test forcing. The only non-triviality in this coupled two-domain problem is jump in stresses across the interface

3.22 $$\left\{-\delta B^{-}+\left[\nabla L^{-}+{\left(\nabla L^{-}\right)}^{T}\right]\right\}\cdot \hat{n}=\left\{-\delta B^{+}+\left[\nabla L^{+}+{\left(\nabla L^{+}\right)}^{T}\right]\right\}\cdot \hat{n}+J,$$

where the third-rank interface stress jump tensor is defined as ${\left(J\right)}_{ikl}=\delta_{ik}n_{l}$. At the sides of the interface cell, we employ periodic boundary conditions, and at the top of the interface cell the zero-stress boundary condition is used, and at the solid structure we have no-slip condition. At the bottom of the interface cell, however, the no-slip condition is used, because there is no L field in the interior. By looking at the governing equations of the test problem (3.20)–(3.22), one can conclude that this test problem is exposed to test forcing at the interface. For example, the problem with the second and third tensor indices being *j*=1 and *k*=3 ($L_{i13}$) corresponds to unit forcing in the *x*-direction at the interface, as shown in figure 5*b*. Thus, this test problem is used to characterize the free-fluid surface stress contribution to the interface velocity. Finally, after solving the coupled two-domain Stokes problem, one can obtain the effective interface slip length, by employing the same volume average $\text{L}=\langle L^{+}\rangle$ above the interface.

Evaluating the slip length tensor at the interface gives

3.23 $$\text{L}\cdot \hat{z}=\left(\begin{array}{ccc} 0.183 & 0 & 0\\ 0 & 0.187 & 0\\ 0 & 0 & 0 \end{array}\right)l\;\text{and}\;\text{L}\cdot \hat{z}=\left(\begin{array}{ccc} 0.191 & 0 & 0\\ 0 & 0.186 & 0\\ 0 & 0 & 0 \end{array}\right)l,$$

for cubic-symmetric and monoclinic-symmetric pore-scale geometries, respectively. In the planar interface case, the only meaningful interface problems for the Navier slip-length are those which relate slip length with velocity shear in interface-normal direction [42]; therefore, we have presented the slip length tensor dot product with unit normal vector $\hat{z}$ normal to the interface with poroelastic material (other entries in this third-rank tensor are zero). The non-zero entries in both matrices corresponds to influence on velocity components *u*_*x*_ and *u*_*y*_ from velocity strains (∂_*z*_*u*_*x*_+∂_*x*_*u*_*z*_) and (∂_*z*_*u*_*y*_+∂_*y*_*u*_*z*_), respectively. For the cubic-symmetric case, both coefficients are similar due to cubic symmetry of the pore-scale geometry. That is, the structure (figure 3*b*) is the same in the *x*- and *y*-directions. The monoclinic-symmetric structure, on the other hand, is different in the *x*- and *y*-directions (figure 4*c*). However, the slip-length coefficients are still similar, because (i) the top cylinder acts exactly the same way in both *x*- and *y*-directions and (ii) the tilted ellipsoid slows down the velocity in both *x*- and *y*-directions similarly in integral sense. For *u*_*z*_ component, there is no contribution in both cases, because the interface-normal penetration velocity is governed by mass conservation alone.

## 4. Poroelastic material response to free fluid vortex above it

The purpose of this section is to exemplify the proposed numerical framework by illustrating the response of the cubic-symmetric and monoclinic-symmetric poroelastic materials from §3 to free fluid vortex above it. In order to create a two-dimensional fluid vortex, we consider a steady low-Reynolds number lid-driven cavity problem, consisting of a free fluid domain *Ω*_*f*_ and a poroelastic domain *Ω*_*p*_. We will validate and characterize the accuracy of the effective continuum description by comparing to a second approach, in which the whole domain is meshed and the flow field as well as the displacement field are resolved at all spatial scales. For the fully resolved numerical studies to be feasible, we do not deform the computational mesh when the microstructure is displaced, which sets an upper limit of displacement we can consider to roughly v≲0.1l. Note that this means we consider a one-way interaction problem, that is the material elasticity does not influence the free fluid, whereas the free-fluid does induce a displacement of the material. The results obtained through this simplification provides new fundamental insight into the physics at the interface between the free-fluid and porous region. It allows us to study how much of the shear stress from the free fluid results in a stronger pore flow and how much of it is borne by the solid. The theory in §§2 and 3 and the numerical implementation of effective equations [46] is valid for two-way coupled problems, but the comparison with fully resolved simulations of such systems we leave for future work.

### (a) Effective continuum description

**Figure 6.**
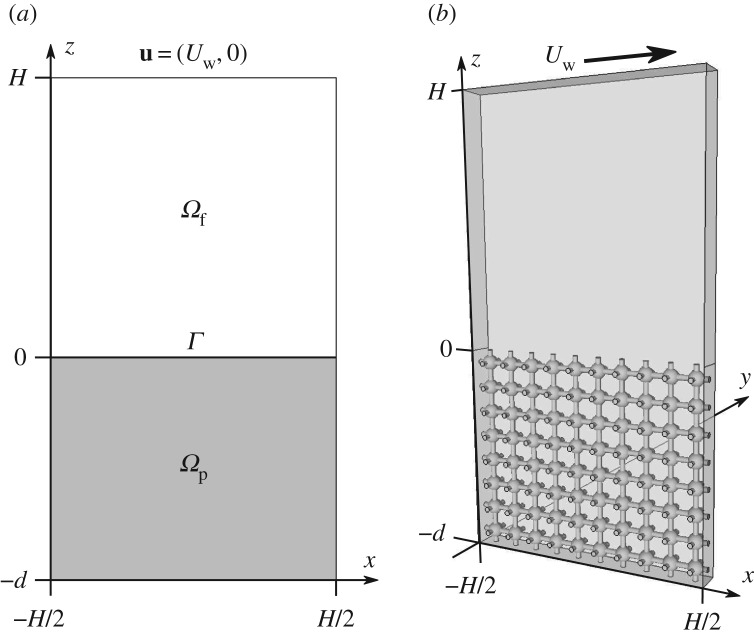
(*a*) A two-domain—consisting of free fluid domain *Ω*_*f*_ and poroelastic domain *Ω*_*p*_—averaged description of the lid-driven cavity flow in two dimensions. (*b*) The lid-driven cavity problem in a three-dimensional setting, which we use for model validation. It is assumed that the flow is periodic in the *y*-direction over a length *l* (one micro-structure). Cubic-symmetric pore-scale geometry is used in this drawing.

As shown in figure 6*a*, the two-dimensional lid-driven cavity has a depth of *H*+*d*, a length of *H* and is infinitely wide. The poroelastic medium is confined to *z*≤0 and −*H*/2≤*x*≤*H*/2. The flow is driven by the top-wall, which moves in the *x*-direction with speed *U*_w_. In *Ω*_*p*_, we solve the effective equations for a poroelastic medium (2.5) and (2.8) with the boundary conditions at the wall (2.9) and at the interface with the free fluid (2.10) and (2.11). For the free fluid in *Ω*_*f*_, we solve equations (2.1) and (2.2) with velocity boundary conditions at the interface with the poroelastic material (2.12) and (2.13). At the side and top walls of the cavity, we impose

4.1 $$\text{u}=0\;\mathrm{at}\;x=\pm \frac{H}{2}\;\mathrm{and}\;\text{u}=(U_{w},0)\;\mathrm{at}\;z=H,$$

respectively. The governing equations are discretized using finite-element method (FEM). The simulation domain along with equations is defined in the FreeFEM++ [48] software using uniform mesh spacing^6^ Δ*s*=0.20*l*. We implement the weak formulation and solve the time-dependent problem in a fully implicit manner, where boundary conditions at the interface are enforced using Lagrange multipliers.^7^ The effective coefficients, such as elasticity tensor and permeability matrix, are taken from §3. To render the validation with the resolved model feasible, we select a moderate scale separation parameter *l*/*H*=0.1.

#### (i) Effective fluid velocity

**Figure 7.**
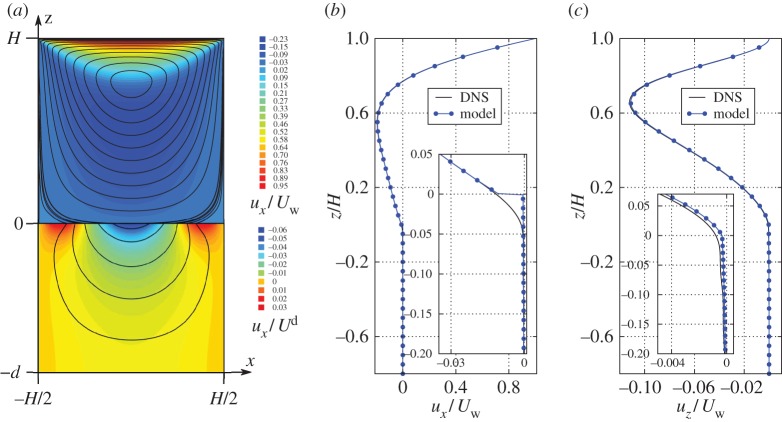
Comparison of flow velocities between effective model of the cavity and the direct numerical simulation of cubic-symmetric pore-scale geometry. In the left frame (*a*), we show model results; the coloured iso-contours corresponds to stream-wise velocity distribution in free fluid and poroelastic material and black lines are flow streamlines. The stream-wise velocity in the poroelastic material is normalized with Darcy's velocity *U*^d^ and in the free fluid it is normalized with upper-wall velocity *U*_*w*_=100*U*^d^. In (*b*), we show the stream-wise velocity variation over the vertical coordinate at *x*=0.1*H*. In (*c*), we show the interface-normal velocity variation over the vertical coordinate at *x*=0.1*H*. The insets in frames (*b*,*c*) show flow results near the interface. (Online version in colour.)

**Figure 8.**
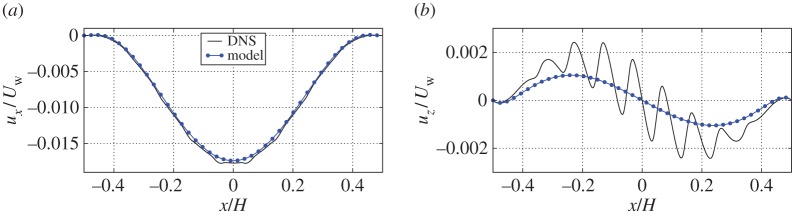
Comparison of flow velocities between effective model of the cavity and the direct numerical simulation of cubic-symmetric pore-scale geometry near the interface. In (*a*), we show slip velocity in the cavity problem near the tip of the solid skeleton at *z*=0.01*H*. In (*b*), we show the penetration velocity at the same coordinate *z*=0.01*H*. (Online version in colour.)

We start by presenting the flow field **u**=(*u*_*x*_,*u*_*z*_) in the full domain (*Ω*_*p*_ and *Ω*_*f*_) for the cubic-symmetric poroelastic medium with porosity *θ*=0.85. Streamlines and isocountours of the streamwise fluid velocity are shown in figure 7*a*. In the free fluid domain, we normalize the results using the top wall velocity *U*_*w*_ (in the simulations we have set *U*_*w*_=100*U*^d^), whereas in the poroelastic domain we normalize the results using Darcy's velocity *U*^d^≡*l*^2^Δ*P*/(*μH*). The moving top wall creates a circulation in the cavity, where the flow for *x*>0 is directed downwards, and upwards for *x*<0. We observe that due to the vortex in the free fluid, there exists a transfer of mass and momentum across the interface. This is characterized in more detail from the slip and infiltration (penetration) velocities very close to the interface (*z*=0.01*H*) in figure 8*a*,*b*, respectively. The streamwise slip velocity (figure 8*a*) has a parabolic shape near the poroelastic medium with its minimum (largest magnitude) velocity at the centre of the cavity. This velocity component is mainly created by the shear of the free fluid, which is the strongest at *x*=0, before it gradually decays when approaching the sides of the cavity. The penetration velocity (figure 8*b*) shows a macroscopic behaviour similar to a sine wave. We observe that for *x*>0, there is a net mass/momentum transport from free fluid region to the poroelastic region, whereas for *x*<0 the net mass flow is in the opposite direction.

Figure 7*a* shows that the flow inside the bed circulates in the same direction as in the free-fluid region. Note that the streamwise velocities in the two domains differ by roughly three orders of magnitude. This gives the impression of a clear discontinuity of streamwise velocity component; figure 7*b*,*c* shows the streamwise *u*_*x*_ and wall-normal *u*_*z*_ velocity profiles for the fixed streamwise position *x*=0.1*H*. For the streamwise component (see inset of figure 7*b*), one can observe that there is a very slow Darcy flow inside the poroelastic material, whereas near the interface there is a jump to the fast slip velocity of the free flow. The leading-order effective equation for flow inside a poroelastic material, which is relative to Darcy's Law (2.5), contains only the pressure contribution from the flow and no viscous fluid stress. As a consequence, the fluid inside the poroelastic layer can only be driven by the normal stress (cannot respond to shear stress), which is the reason why the velocity jump arises. In figure 7*c*, we see that wall-normal component *u*_*z*_ is continuous across the interface, which follows from mass conservation.

We compared the homogenized model results between cubic-symmetric poroelastic material (*θ*=0.85) and monoclinic-symmetric poroelastic material (*θ*=0.86) and did not observe any significant difference in flow velocities, despite the different levels of anisotropy in the pore microgeometries. The reason for this outcome is the fact that the introduced anisotropy results only in higher order corrections of permeability tensors (3.11), (3.13) and (3.19) and Navier-slip tensors (3.23).

#### (ii) Effective solid displacement

**Figure 9.**
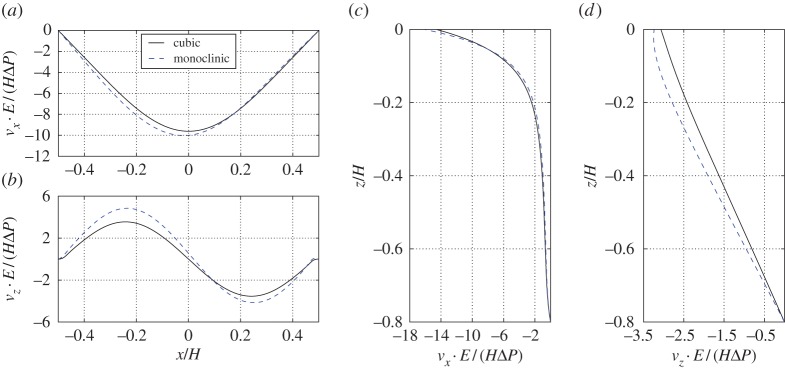
Comparison between effective solid displacement data for cavity with poroelastic bed built from cubic-symmetric and monoclinic-symmetric geometries. In (*a*), we show horizontal displacement near the tip of the solid skeleton at *z*=−0.05*H*. In (*b*), we show the vertical displacement at the same coordinate *z*=−0.05*H*. In (*c*), we show the horizontal displacement variation over the vertical coordinate at *x*=0.15*H*. In (*d*), we show the vertical displacement variation over the vertical coordinate at *x*=0.15*H*. It is estimated that the displacements have to be v≲0.1l in order for the current implementation to be reliable despite the non-deforming mesh. (Online version in colour.)

In figure 9*a*,*b*, we show displacement *v*_*x*_ and *v*_*z*_ along *x*-coordinate at a fixed *z*=−0.05*H* for both cubic-symmetric and monoclinic-symmetric pore-scale geometries. The displacement fields are normalized^8^ using length scale *H*Δ*P*/*E*. Moreover, the *z*-coordinate is chosen to correspond to the centres of connecting cylinders. In this way, the result can easily be compared to fully micro-resolved simulations (see next section), for which it is not straight-forward to define displacement fields in the fluid part of the pores. For both microstructure geometries, the horizontal displacement *v*_*x*_ near the interface (figure 9*a*) reveals a similar behaviour as for the slip velocity, i.e. the displacement has parabolic shape with maximum magnitude at the centre of the cavity. The horizontal displacement is in the direction of the slip velocity directly above the interface and is caused by the shear stress induced by the overlying flow vortex in the cavity. The vertical displacement *v*_*z*_ near the interface (figure 9*b*), on the other hand, shows a very similar behaviour to that of the penetration velocity, i.e. the displacement has a sine-like shape. We thus observe that the solid displacement is complying to the tangential and normal fluid velocities near the interface.

In figure 9*c*,*d*, we show *v*_*x*_ and *v*_*z*_ along the *z*-coordinate at a fixed position *x*=0.15*H*. For the horizontal component *v*_*x*_ (figure 9*c*) two regions can be identified, where the displacement decreases at different rates. The region down to z≳−0.2H, for which the decay is very fast, is determined by the shear stress at the interface. Below z≲−0.2H, it is the slow Darcy flow that induces the small displacement. The vertical displacement (figure 9*d*), on the other hand, is entirely governed by the slow penetration velocity inside the medium or the interface-normal stress at the interface, depending on which one is the dominating effect.

We note that the horizontal displacement (figure 9*a*) has slightly larger magnitude and is skewed for the monoclinic compared to the cubic material. This difference can be explained by a slightly smaller compression elasticity coefficient *C*_*xxxx*_ (4.668<4.792) and by the existence of an elasticity coefficient *C*_*xzxx*_ for the monoclinic material (see §3a(iii)), which relates the strain of *v*_*x*_ in the *x*-direction with the stress in the interface-normal direction *z* on the plane with a fixed *x*-coordinate. The vertical displacement (figure 9*b*) is also larger in magnitude and skewed for the monoclinic geometry. The larger amplitude can again be explained by the difference in compression elasticity coefficient *C*_*zzzz*_ (4.031<4.792), whereas the skewness can be attributed to *C*_*xxxz*_, which provides a coupling between the interface-normal stress of the free fluid to strain of the vertical displacement *v*_*z*_ in the *x*-direction. We thus conclude that an anisotropy in the microstructure breaks the symmetric behaviour of the solid displacement in the macroscale due to the additional cross terms (such as *C*_*xxxz*_) in the effective elasticity tensor.

### (b) Validation to fully microscale-resolved simulations

In order to validate the effective model, a three-dimensional lid-driven cavity problem is considered (same depth of *H*+*d* and length *H* as before), where the flow is periodic in *y*-direction over length *l*, which is set by the pore structure. Therefore, it is sufficient to consider the domain shown in figure 6*b* (spanning over one microstructure in *y*-direction) for direct numerical simulations (DNS).

The domain shown in figure 6*b* is defined using GMSH software [47] and meshed using spacing Δ*s*_1_=0.04*l* at the solid skeleton and Δ*s*_2_=0.16*l* at the boundaries of the cavity.^9^ We import the generated mesh into FreeFEM++ [48], in which we define and solve the governing equations (2.1)–(2.3) with boundary conditions (2.4) and (4.1). At the walls, we use no-slip velocity for fluid and zero displacement for solid structure. To simplify the numerical task, we assume a steady flow and neglect inertial effects, which simplifies the Navier–Stokes equations (2.1) and (2.2) to the linear Stokes equations. We use Taylor-Hood (P2+P1) elements for the Stokes system, and quadratic elements (P2) for the solid skeleton linear elasticity system (2.3). The resulting linear algebraic system is solved using a GMRES iterative linear solver up to a relative tolerance *e*=10^−10^. For the solid skeleton, we choose the isotropic material that was used as the starting point for the computation of effective properties in §3 (Poisson's ratio *ν*=0.3 and Young's modulus *E* unspecified). We assume that deformations are small enough such that the computational mesh can be kept static; this limits the range of length scales $\bar{l}=H\Delta P/E$—based on Young's modulus *E*, large scale length *H* and pressure difference Δ*P*—that can be considered.

#### (iii) Fully resolved fluid flow

**Figure 10.**
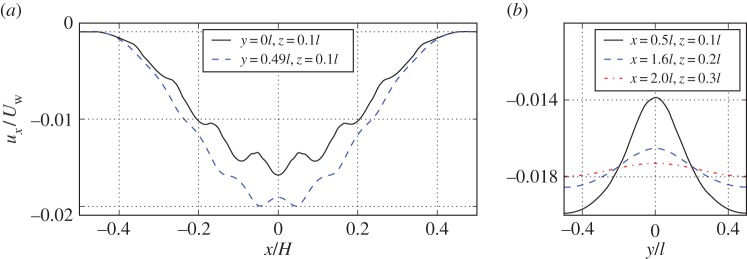
Plots of raw fluid simulation data for cavity with cubic-symmetric poroelastic bed. In (*a*), we show slip velocity in the cavity problem near the tip of the solid skeleton (*z*=0.01*H*). We choose to sample the obtained results at the centre of the three-dimensional slice (*y*=0.0*l*) and near the periodic boundary (*y*= 0.49*l*). In (*b*), we show the stream-wise velocity variation in the periodic direction at points, where the average velocity value is roughly the same. Coordinates of the line probe are *x*=0.05*H*, *z*=0.01*H*; *x*=0.16*H*, *z*=0.02*H* and *x*=0.20*H*, *z*=0.03*H*. (Online version in colour.)

In figure 10*a*, we show the resolved stream-wise velocity (slip velocity) near the interface at a distance *z*=0.01*H* and coordinates *y*=0 and *y*=0.49*l*. The poroelastic bed in this case is built using cubic-symmetric geometry. We observe that the slip velocity is slightly slower at the centre of the cavity slice (*y*=0) compared to the *xz*-plane near the periodic boundaries (*y*=0.49*l*). This is because the bulk of the solid material (sphere) is located at the centre of each volume element, therefore the surrounding fluid is slowed down more than the fluid near the cylindrical obstructions close to the periodic boundaries. The stream-wise velocity distribution in the *y*-direction at three *x*- and *z*-coordinates is shown in figure 10*b*. Moving away from the poroelastic material (increasing *z*-coordinate) leads to a rapid dissipation of velocity variation; that is, the flow velocity approaches a constant value with respect to the *y*-coordinate. We continue by averaging the DNS quantities in the *y*-direction as

4.2 $$\bar{f}(x,z)=\frac{\int_{-l/2}^{l/2}I_{d}f(x,y,z)\;dy}{\int_{-l/2}^{l/2}I_{d}\;dy},$$

where *f* denotes any of *u*_*x*_, *u*_*y*_, *u*_*z*_, *p*, *v*_*x*_, *v*_*y*_ and *v*_*z*_. The function *I*_*d*_ is an indicator function, which is *I*_*d*_=1 in the domain, where field *f* is defined, and *I*_*d*_=0 elsewhere. This average is also known as intrinsic average [30], because it is normalized by fluid or solid volumes separately. For convenience, we omit the ‘bar’ notation further on.

The average slip velocity and penetration velocity variations over the *x*-coordinate sampled at *z*=0.01*H* are shown in figure 8*a*,*b*, respectively, together with the effective model curves; one can conclude that the macroscale model is accurate. Note that the effective model is fully non-empirical without any fitting parameters. The penetration velocity (figure 8*b*), shows micro-scale oscillations over one pore-scale structure in the resolved simulations, which are by construction not captured by the effective equations. The penetration velocity is somewhat under-predicted by the effective model, which may be an indication of a pressure jump [43,44], not modelled in the current work.

#### (iv) Fully resolved solid displacement

**Figure 11.**
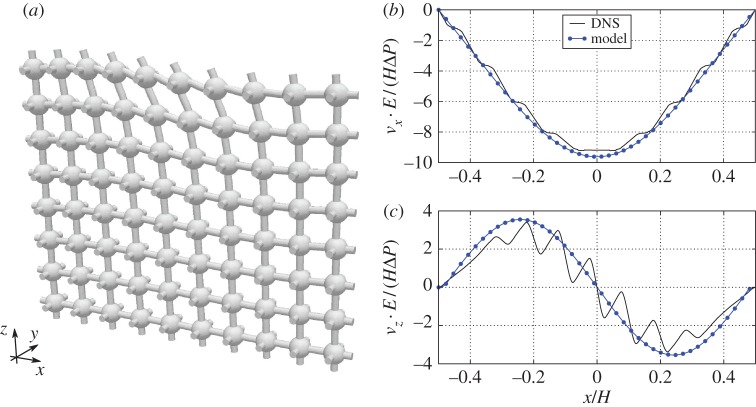
In (*a*), we show a deformed shape of the solid skeleton built using cubic-symmetric pore-geometry, exposed to the stress from the surrounding flow. The displacements are magnified in order to make the distortion of the skeleton visible. In (*b*), we show horizontal displacement near the tip of the solid skeleton (*z*=−0.5*l*). In (*c*), we show the vertical displacement at the same coordinate *z*=−0.5*l*. Both effective model results and fully resolved results are compared. (Online version in colour.)

Figure 11*a* shows the deformation of the cubic-symmetric structure due to the free fluid vortex above. The figure serves only as an illustration, where the solid structure is displaced after the computation, since our implementation is restricted to static meshes. The solid displacement obtained from DNS is compared to the effective model over a horizontal slice at *z*=−0.05*H*=−0.5*l* in figure 11*b*,*c*. Similarly as for the fluid velocity, we observe that the microscale features of the displacement field are not captured by the leading-order effective model, but that the macroscale behaviour is in good agreement.

**Figure 12.**
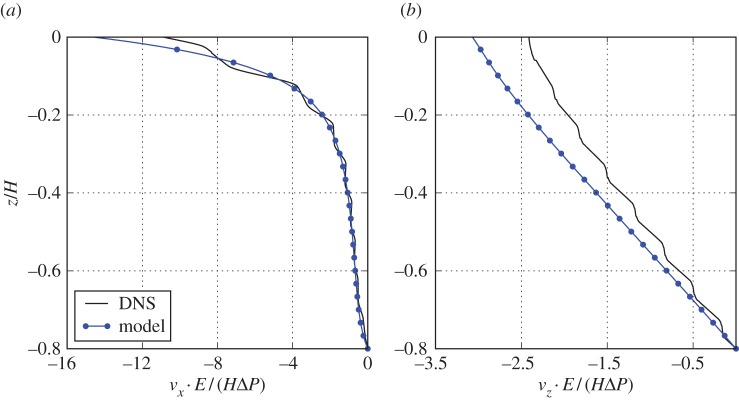
In (*a*), we show horizontal displacement of the poroelastic medium build from cubic-symmetric pore geometry in depth at coordinate *x*=0.15*H*. In (*b*), we show the vertical displacement at the same coordinate *x*=0.15*H*. Both homogenized model results and fully resolved results are compared. (Online version in colour.)

Figure 12*a* compares the horizontal displacement along the *z*-coordinate. This shows that the effective model overestimates the horizontal displacement close to the interface, but is very accurate below z≲0.05H. This indicates that the inaccuracy is caused by the interface stress boundary condition (2.11). The vertical displacement (figure 12*b*) is overestimated over the entire depth of the cavity, which suggests that the vertical displacement is mostly governed by the stress at the interface between the free fluid and the poroelastic medium. Despite the inaccuracy at the interface, the model captures the essential qualitative features of the displacement field, including the two different regions of decay of the horizontal displacement. The seemingly good agreement of the horizontal displacement in figure 11*b* arises due to the micro-scale variations of the DNS solution, as shown in figure 12*a*.

Our results show that the free-fluid shear stress transfer to the poroelastic material shear stress is a macroscopic phenomenon. The influence of the interface stress is ranging over a number of pore structures; the shear region in figure 12*a* extends over distance of *z*≈0.3*H* containing 2–3 unit-cell structures. However, the free-fluid shear stress transfer to pore fluid shear stress is a microscopic phenomenon. The influence of the stress is ranging over less than one pore structure; the region over which velocity decays to Darcy's velocity in figure 7*b* extends over distance of *z*≈0.05*H*=0.5*l* containing only half of one unit-cell structure. Over this short distance, all the fluid shear stress is transferred over to the solid skeleton, thus contributing to large shear region in figure 12*a*.

**Figure 13.**
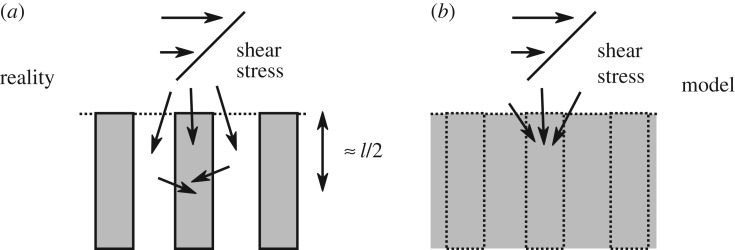
In (*a*), we illustrate how the free fluid shear stress (sketched with linearly increasing horizontal velocity profile) in reality is transferred to both solid skeleton (sketched as grey obstacles) and to pore fluid. Owing to viscous dissipation, the pore fluid shear stress is then transferred to solid skeleton over microscale length ≈*l*/2. In (*b*), we show that in the model equations all of the free fluid shear stress is transferred directly into the solid skeleton at the artificial interface between poroelastic region and free fluid region.

As a final comment, the effective properties (**C**,***α*** and E) of the interior can be used at the interface for the stress boundary condition (2.11). The reason is that the shear stress from the free fluid is eventually borne only by solid skeleton. As illustrated in figure 13*a*, from the microscopic—or ‘reality’— point of view, the free fluid shear stress is transferred to both solid skeleton and pore fluid. However, roughly a half of a microstructure below the interface, the shear stress of the pore fluid is transferred to the surrounding solid skeleton via viscous friction. Therefore, the elasticity tensor from the interior is a reasonable estimate for the elasticity tensor at the interface. In other words, the model (figure 13*b*), in which free fluid shear stress is transferred directly to solid skeleton only, is a reasonable approximation. This is, however, not the case for the velocity boundary conditions (2.12) and (2.13), for which the micro-scale viscous dissipation has to be modelled by Navier-slip term (**L**); otherwise, as shown by Lācis & Bagheri [42], table 2, the predicted interface velocity would be around 1/*ϵ* times smaller compared with DNS. This is due to the fact that the pore fluid undergoes a rapid acceleration caused by the free fluid shear at the interface, which renders the interior velocity a very inaccurate estimate for the interface velocity.

### (c) Capturing anisotropic effects with the effective model

**Figure 14.**
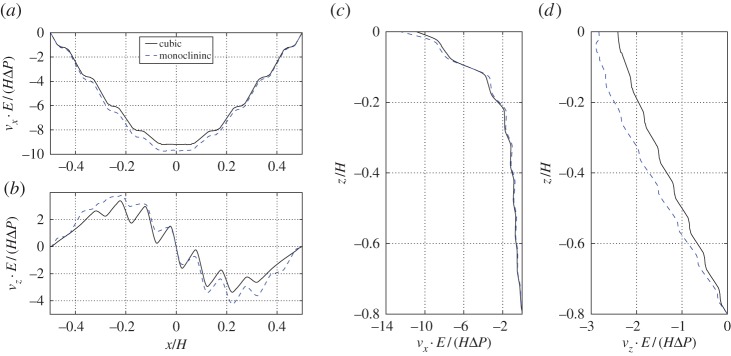
Comparison between DNS solid displacement data for cavity with poro-elastic bed constructed using cubic-symmetric and monoclinic-symmetric geometries. In (*a*), we show horizontal displacement near the tip of the solid skeleton at *z*=−0.05*H*. In (*b*), we show the vertical displacement at the same coordinate *z*=−0.05*H*. In (*c*), we show the horizontal displacement variation over the vertical coordinate at *x*=0.15*H*. In (*d*), we show the vertical displacement variation over the vertical coordinate at *x*=0.15*H*. (Online version in colour.)

The displacement fields of the two geometries (cubic-symmetric and monoclinic-symmetric) obtained from DNS are compared in figure 14. For the monoclinic-symmetric geometry, the horizontal displacement is roughly 6% larger, and the vertical displacement is roughly 10% larger (compared to cubic-symmetric geometry). This difference cannot be explained by the change of porosity only (around 1%); therefore, the reason must be the introduced anisotropy of the pore geometry. Moreover, in the horizontal and the vertical displacement fields one can observe a small asymmetry between left and right half of the cavity, which is a sign of symmetry breaking due to anisotropic effects.

In §4a(ii), a difference was also observed between the two microstructures in the effective model, which was explained by comparing the entries of the effective elasticity tensor. Figure 14, obtained using fully resolved simulations, can be compared with the corresponding figure obtained from the effective model (figure 9). We observe that the relative change between those two geometries is accurately captured by the effective model. This indicates that using the interior effective elasticity, when imposing stress boundary conditions at the interface, provides an effective model that is practically useful in capturing differences in the macroscale, caused by anisotropy within the microstructure.

## 5. Discussion

The numerical framework presented here is based on first solving a set of microscale problems from §3 to compute the effective tensors (**C**, **K**, ***α***, **K**^if^ and **L**), followed by solving the macroscopic effective equations coupled through interface conditions to the free fluid (§2). The equations formed in both these steps stem from multi-scale homogenization approach and rest on a number of assumptions, similarly as explained in the literature [50,51] for the method of volume averaging. These assumptions restrict the range of physical parameters that we are able to model using effective continuum theory. It is therefore essential to understand if the admissible range of parameters are relevant for solving physical problems that arise in nature and engineering. In this section, we discuss the necessary assumptions used in the development of the current framework and how they relate to physical constraints.

The first and foremost prerequisite is scale separation. Based on the numerical tests in the literature [42] and §4, we have determined the practical limit on the scale separation parameter

5.1 $$\epsilon =\frac{l}{H}≲0.1,$$

which is less restrictive than the asymptotic limit l/H→0. In other words, one does not need to be in the asymptotic limit in order to apply the current framework. We compared the effective model results with predictions from fully resolved simulations in §4 and observed good agreement with respect to the flow and the displacement fields for the scale separation parameter *l*/*H*=0.1. It is interesting to point out that Auriault [52] has shown the next order corrections to the Darcy's Law to be zero for macroscopically homogeneous porous media, therefore the Darcy's Law holds well also in the case of poor scale separation. This would explain the good agreement between the model and DNS for the flow velocity. It is possible that similar conclusion could be drawn also from the correctors of the elasticity problem. It is likely that condition (5.1) can be further relaxed if an introduced error of given magnitude is acceptable. It should be possible to estimate the upper bound on scale separation parameter by analysing the introduced error, similarly as Drugan & Willis [53] have done for heterogeneous elastic composite. More detailed investigation of the upper bound for scale separation parameter is left as an open question.

We assume that the pore Reynolds number is smaller than one, i.e.

5.2 $$\frac{\rho_{f}U^{d}H}{\mu }\leq O(1).$$

Here, recall that the *U*^d^≡*l*^2^Δ*P*/(*μH*) is the definition of seepage velocity. The above assumption is a good one for poroelastic materials that are densely packed, resulting in a slow flow through the pores, which can be described using steady linear Stokes equations—such as those formulated for **K**, **K**^if^ and **L** in equations (3.9)–(3.10), (3.15)–(3.18) and (3.20)–(3.22). For larger pore Reynolds numbers, the microscopic problems will become nonlinear, which renders the current multi-scale approach, based on linear decomposition, unfeasible. A possible workaround is to use some kind of linearization, similarly as done by Zampogna & Bottaro [36].

Next, we make an assumption on how the macroscopic free fluid time scale Δ*τ* that force the poroelastic bed is related to the microscopic time scale *l*/*U*^d^ inside the bed. Specifically, the frequency 1/Δ*τ*, at which the free fluid interacts with the poroelastic bed, has to satisfy

5.3 $$\frac{1}{\Delta \tau }\;\frac{l}{U^{d}}\leq O(1).$$

This essentially states that the changes in the free fluid must be slower compared with the time a fluid parcel needs to travel a pore length *l*. Thus, from the microscale viewpoint, the external macroscopic forcing is slow, which in turn has the consequence that the corresponding microscale problems (3.9)–(3.10), (3.15)–(3.18) and (3.20)–(3.22) are steady. If external forcing frequency is higher than *U*_*d*_/*l*, the Stokes problems (3.9)–(3.10), (3.15)–(3.18) and (3.20)–(3.22) will become time-dependent, which in turn would require solving convolution integrals in order to take into account the time history, see Mei & Vernescu [13], eqn 6.6.11.

Another restriction of the current method is on the relative size of the characteristic normal stresses of the flow and the solid skeleton. That is, we assume that the macroscale global Δ*P* relates to the characteristic Young's modulus of the solid skeleton as follows:

5.4 $$\frac{\Delta P}{E}\leq O(\epsilon ).$$

This assumption holds in many engineering configurations, where elastic properties of materials are often of order MPa or GPa, while the pressure difference generated by moving fluids is commonly of order kPa (in wind tunnel experiments, for example). It also holds for many biological systems; the elasticity moduli are of order MPa or kPa [54–56], where biological materials are often exposed to much slower fluid flows [57] and consequently smaller fluid forces.

The final requirement is that inertial effects of the solid skeleton at the microscopic level are small. We expect that the pore-flow is sufficiently slow such that inertial effects of the solid skeleton in the microscale are not excited. This implies a relationship among the characteristic macroscopic pressure, the solid density and characteristic time scale:

5.5 $$\frac{\rho_{s}lH}{\Delta P\Delta \tau^{2}}\leq O(1).$$

This results in linear, time-independent microscale solid test problems (3.3)–(3.4) and (3.6)–(3.7). If this restriction is not obeyed, the test problems should be complemented using pore-scale inertial effects.

Note that the above assumptions are not unique and in order to understand mathematically why these particular choices are made, we refer the reader to the electronic supplementary material, appendix A. At the end, we want to understand whether the resulting effective equations governing the poroelastic bed can be used to describe the length and time scales that are physically relevant. In order to do so, we compare restrictions, involving the external macroscopic forcing time scale Δ*τ* (5.3) and (5.5), with the intrinsic poroelastic time scale Δ*τ*_*p*_ and the time scale Δ*τ*_*i*_ related to the speed of waves in the effective bed. The former time scale Δ*τ*_*p*_ characterizes the time for the pressure field to equilibrate via fluid transport in the medium and therefore determines how fast the poroelastic bed can respond to external forcing [58]. The latter time scale Δ*τ*_*i*_ characterizes the time for wave propagation, and therefore also the time it takes for information to propagate through the effective bed. Following Skotheim & Mahadevan [58], the time scales are given by

$$\Delta \tau_{p}∼\frac{\mu H^{2}}{\left(kC_{\mathrm{eff}}\right)}\;\mathrm{and}\;\Delta \tau_{i}∼H\sqrt{\frac{\rho_{s}}{C_{\mathrm{eff}}}},$$

where *k* is the characteristic permeability and *C*_eff_ is the characteristic effective elasticity coefficient. These time scales can be compared to the assumptions discussed above using estimates *k*∼*l*^2^ and *C*_eff_∼*θ*_s_*E*, where *θ*_s_=1−*θ* is the solid volume fraction. Using assumptions (5.3) and (5.4) from the current work, we then arrive to inequality Δ*τ*≥*θ*_s_Δ*τ*_*p*_. This shows that the macroscopic driving force time scale can be of the same order as the poroelastic material time scale, therefore the model derived in the current work allows for description of poroelastic effects in the medium. Furthermore, using assumptions (5.4) and (5.5), we arrive to inequality $\Delta \tau \geq \sqrt{\theta_{s}}\Delta \tau_{i}$. This indicates that the model explained in this work allows for description of travelling waves through poroelastic medium. To sum up, the current multi-scale approach should allow for the description of problems where Δ*τ* is of the order of Δ*τ*_*p*_ (to capture strong fluid–elasticity interaction [58]) and also of the order of Δ*τ*_*i*_ (to capture travelling waves or elastic instabilities).

## 6. Conclusion

We have considered the problem of a free-fluid interacting with a poroelastic bed, by deriving and validating an effective continuum model for the bed and its interface with the above free fluid. Although the effective equations of the interior of the bed are well established, their coupling to a non-trivial vortical free fluid through a set of interface conditions have not been considered and validated before. The imposed interface conditions are (i) the velocity boundary condition for the free fluid; (ii) the pressure continuity boundary condition for the pore-pressure and (iii) the stress continuity boundary condition for the displacements of poroelastic media. The first two conditions have been derived from first principles using certain assumptions, while the stress boundary condition is postulated *a posteriori*. In particular, for condition (iii) the interior effective parameters (**C** and ***α***) were used, whereas for condition (i) the interface permeability and the Navier-slip tensor were computed. This asymmetry in the treatment of the boundary condition is motivated by the fact that for the velocity boundary condition—which is needed to solve for the free-fluid—the transfer of shear-stress to pore flow requires a new Navier-slip tensor **L**, because Darcy's Law cannot accommodate any shear. Therefore, the interfacial velocity condition requires a special treatment. For the stress boundary condition—which is needed to solve for the displacement in the bed—both normal and tangential stresses induced by the free flow above can be matched by the corresponding stresses of the solid. We have shown that using condition (iii), based on the interior coefficients, provides a satisfactory model; on the one hand, it does result in a—small, but not insignificant—discrepancy with the fully microscopic simulations that we use as a validation; on the other hand, it captures the effects of small changes in the microstructure anisotropy correctly and predicts the overall behaviour in a physically consistent and controllable manner. We thus believe that this approach for modelling the interaction of poroelastic beds with freely moving fluids is a viable framework for engineers. The practical limits of the derived model have been discussed, where we show that the proposed model can be employed for any physically relevant poroelastic material. The corresponding codes of the numerical implementation used in the present work have been released as an open-source software [46].

In the future, we will further improve the model by treating the stress boundary condition using appropriate interface cells. We also want to validate the proposed model for unsteady flows, where inertia is not negligible. Finally, the next step for the model development is to extend it to significant surface deformations, that would require moving the interface and porosity variation in space.

## Footnotes

1 The discussion can be found in the electronic supplementary material, appendix B, after equation (B 19).

2 We use mesh spacing Δ*s*_1_=0.025 *l* at the solid skeleton and Δ*s*_2_=0.10*l* at the boundaries of the unit cell. We have also carried out the simulation on coarser meshes (Δ*s*_1_=0.05*l* and Δ*s*_2_=0.20*l*; Δ*s*_1_=0.10*l* and Δ*s*_2_=0.40*l*) and, by observing the convergence of results, we set entries containing only numerical noise to zero.

3 The difference of 2×10^−5^ between the first and second diagonal element is attributed to numerical error that could be caused by, for example, imperfections in the generated mesh.

4 For explicit relationships obtained using multi-scale expansion, see the electronic supplementary material, appendix C.

5 Similarly as in interior simulations, we carry out investigations using three different resolutions, Δ*s*_1_=0.05*l* and Δ*s*_2_=0.20*l*, Δ*s*_1_=0.0625*l* and Δ*s*_2_=0.2500*l* and Δ*s*_1_=0.10*l* and Δ*s*_2_=0.40*l*. The interface cell is chosen to encompass three structures below the interface and is extending for the same distance in the free fluid.

6 We have investigated results using half of the mesh spacing Δ*s*=0.10*l* and observed that the slip velocity changed by 0.4% and the horizontal displacement near the interface changed by 0.8% at the centre of the cavity.

7 Exact weak form can be found in documentation of the open-source software [46].

8 Note that due to the linearity of governing equations, the results are valid for any chosen Young's modulus, as long as the deformation is small enough (v≲0.1 l) to neglect the mesh deformation and Poisson's ratio is *ν*=0.3. The dimensional normalization is obtained in the electronic supplementary material, appendix A.

9 We have carried out same simulations using mesh spacings Δ*s*_1_=0.05*l* and Δ*s*_2_=0.20*l*. The change in fluid slip velocity was everywhere below 0.3%. The change in horizontal solid displacement was everywhere below 5.5%, with maximum at the centre of the interface.

## Data accessibility

The core of the codes used for producing the plots in the current paper are available as open source software in the Github repository [46]. The same repository contains the flow and displacement fields of DNS simulations presented in this paper.
